# *BjuFKF1_1,* a Plant-Specific LOV Blue Light Receptor Gene, Positively Regulates Flowering in *Brassica juncea*

**DOI:** 10.3390/plants15020270

**Published:** 2026-01-15

**Authors:** Jian Gao, Keran Ren, Chengrun Wu, Qing Wang, Daiyu Huang, Jing Zeng

**Affiliations:** 1 College of Agriculture and Biotechnology, Lishui University, Lishui 323000, China; gaojian_genomics@163.com; 2 College of Agronomy and Biotechnology, Southwest University, Chongqing 400716, China; 3 School of Advanced Agriculture and Bioengineering, Yangtze Normal University, Chongqing 408100, China; 4 College of Biology and Food Engineering, Chongqing Three Gorges University, Chongqing 404100, China

**Keywords:** *B. juncea*, *BjuFKF1_1*, overexpression line, early-flowering

## Abstract

Stem mustard (*Brassica juncea* var. tumida *Tsen* et Lee) is an important economic vegetable in China. Premature bolting induced by temperature fluctuations has become a major cultivation constraint. Photoreceptors (PHRs) serve as critical photosensor proteins that interpret light signals and regulate physiological responses in plants. In this study, five core PHR families, namely *F-box-containing flavin binding proteins* (*ZTL/FKF1/LKP2*), *phytochrome* (*PHY*), *cryptochrome* (*CRY*), *phototropin* (*PHOT*) and *UV RESISTANCE LOCUS 8* (*UVR8*) were identified in *Brassica* species. RNA-seq analysis revealed their expression patterns during organogenesis in *B. juncea*. Seven candidate PHRs were validated by qRT-PCR in *B. juncea* early-bolting (‘YA-1’) and late-bolting (‘ZT-1’) cultivars. *Agrobacterium*-mediated *BjuFKF1_1* overexpression (OE) lines resulted in significantly earlier flowering under field conditions. Histochemical GUS staining indicated that *BjuFKF1_1* was expressed in seedlings, leaves, flower buds and siliques. Transcript analysis revealed that the expression level of *BjuFKF1_1* was up-regulated in all tissues at both the vegetative and reproductive stages, whereas the expression of BjuFKF1_1 interacting protein-encoding genes were down-regulated in flowers. Under blue light, genes encoding interacting proteins (*BjuCOL5*, *BjuSKP1*, *BjuCOL3*, *BjuAP2*, *BjuAP2-1* and *BjuLKP2*) were up-regulated in flower buds, whereas *BjuCOL* and *BjuPP2C52* were down-regulated in flowers. Developmental stage analysis revealed the up-regulation of five (*BjuAP2*, *BjuCOL3*, *BjuCOL5*, *BjuAP2-1* and *BjuLKP2*) and four (*BjuCOL*, *BjuCOL5*, *BjuAP2* and *BjuLKP2*) interaction protein-encoding genes during the reproductive stage under white and blue light, respectively. These findings elucidate the role of *BjuFKF1_1* in flowering regulation and provide molecular targets for *B. juncea* bolting-resistant variety breeding.

## 1. Introduction

*B. juncea* can be commercially processed into zhacai and faces significant yield and quality constraints in major production regions (Zhejiang, Sichuan and Chongqing provinces). As the dominant processing product of this crop, zhacai is recognized as one of the world’s three most renowned pickled vegetables. In *Brassica* crops, bolting is triggered by environmental cues such as vernalizing temperatures and photoperiod, which lead to early flowering and further reduce the annual yield and commercial value by 15% [1,2,3]. Therefore, controlling bolting presents a fundamental constraint for *B. juncea* cultivation breeding, and development of bolting-resistant varieties to expand high altitude cultivation in Chongqing’s mountainous regions through advanced breeding technologies is imperative.

In recent years, extensive research has been conducted on model plants to elucidate the molecular basis of the floral transition and six key regulatory pathways—the photoperiod (mediated by photoreceptors) [4,5], vernalization [4,6], ambient temperature [7], gibberellins (GAs) [8], autonomous [9] and age-dependent pathways [10]—have been reported, in which the photoreceptors are the major sensors of light and play important roles in circadian oscillation, acting as important regulators of both floral transitions and ambient temperature sensing [11,12]. Several photoreceptors have been identified, as mentioned below, such as phytochromes A-E (phyA-E), cryptochromes (CRYs), phototropins (PHOTs), ZEITLUPE (ZTL) family members *ZTL*, light/oxygen/voltage (*LOV*) *KELCH PROTEIN2* (*LKP2*) and *FLAVIN-BINDING KELCH REPEAT-BOX1* (*FKF1*), as well as *UVR8* [13]. Five types of phytochromes (from *PHYA* to *PHYE*) and cryptochromes (*CRY1* and *CRY2*) have been identified in *Arabidopsis* and cereals, respectively [14,15,16,17]. Researchers have shown that these phytochromes and cryptochromes affect flowering regulations related to photoperiodism and light’s spectral composition. PhyA, phyB, CRY1 and CRY2 have been shown to physically interact with the COP1-SPA complex. Under red/far-red and blue light conditions, this complex disrupt its E3-ligase activity, thereby promoting photomorphogenesis through accumulation of positively acting transcription factors (e.g., *HY5* and *HFR1*) [1,18,19,20,21,22,23,24].

PHOTs, as plasma membrane-associated kinases, have been reported as UV/blue light-absorbing cofactors through binding to oxidized flavin mononucleotide (FMN). In addition, two light, oxygen, or voltage-sensing domains (LOV1 and LOV2) were identified at *N*-terminus [25,26]. Phototropin kinase activity and receptor auto-phosphorylation are primarily activated by LOV2 [27,28]. Moreover, multiple phosphorylation sites have been shown to be important for signaling within PHOT1 and PHOT2, whereas kinase-inactive variants of phot1 and phot2 are non-functional [29,30,31]. Recently, studies found that *CONVERGENCE OF BLUE LIGHT AND CO*_2_ *1* (*CBC1*), *BLUE LIGHT SIGNALING 1* (*BLUS1*), *ATP-BINDING CASSETTE B19* (*ABCB19*) and *PHYTOCHROME KINASE SUBSTRATE 4* (*PKS4*) were the substrates of PHOT1-kinase, of which *CBC1* and *BLUS1* were found to be involved in blue-light-induced stomatal opening, while *ABCB19* and *PKS4* modulated hypocotyl phototropism [27,32,33,34,35].

*FKF1*, a type of plant-specific blue light receptor, has been reported to promote flowering. It contains three conserved domains: namely a light, oxygen and voltage (LOV) domain, a subfamily of PAS domain located in its *N*-terminus and an F-box domain in its *C*-terminus. *FKF1* functions in protein degradation by forming a SKP1/CUL/F-box (SCF)-type E3 ligase complex through its F-box domain [36]. In *Arabidopsis*, *AtFKF1* mutants exhibit a photosynthetic and a slight cycle rhythm deficit phenotype, suggesting that *AtFKF1*’s functions are regulating plant cycle rhythm and flowering [5,37]. Further studies found that *FKF1* functions primarily through protein–protein interactions and post-translational modifications. Specifically, the FKF1-GI complex responds to blue light and recruits SKP1 to form an SCF ubiquitin ligase complex, which targets *CDF* transcription factors for proteasomal degradation [38]. Consequently, this degradation relieves the repression of *Constants* (*CO*), allowing for the activation of *FT* (*Flowering locus T*) expression and promotion of flowering [39,40,41]. Additionally, the FKF1-COP1 interaction further modulates CO stability, adding another regulatory layer to photoperiodic flowering [42,43]. *UV8* was found to be involved in UV-B stress susceptibility. It binds to the CONSTITUTIVELY PHOTOMORPHOGE COP1NIC 1 (COP1)-SUPPRESSOR OF PHYA-105 (SPA) protein complex and forms an E3 ubiquitin ligase complex. This E3 ubiquitin ligase complex was involved with a number of proteins (notably ELONGATED HYPOCOTYL 5 (HY5) transcription factor) and regulated many gene targets of *UVR8* signaling [44,45,46,47]. In addition, UVR8 destabilized PHYTOCHROME INTERACTING PROTEIN 5 (PIF5) by binding to the COP1 protein to contribute to the suppression of extended growth by UV-B [47]. In our previous study, we confirmed that BjuFKF1_1 interacts with BjuLKP2, BjuPP2C52 (phosphatase 2C52), BjuCOL (CONTANS-like), BjuCOL3 (CONTANS-like 3), BjuCOL5 (CONTANS-like 5), BjuAP2 (APETALA 2), BjuAP2-1 (APETALA 2-1) and BjuSKP1f [48,49].

In this study, five major *PHR* gene families, namely F-box-containing flavin binding proteins (*ZTL/FKF1/LKP2*), *PHY*, *CRY*, *PHOT* and *UVR8,* were identified in *Brassica* species. The expression patterns of these genes in *B. juncea* were determined via RNA-seq data during organ development and tumorous stem development. Moreover, seven candidate PHRs were validated by qRT-PCR in ‘YA-1’ and ‘ZT-1’ cultivars. *BjuFKF1_1* OE lines were subsequently generated and phenotypic observation revealed that the *BjuFKF1_1* OE lines exhibited significantly early flowering in the field. Furthermore, qRT-PCR results revealed that the expression levels of all BjuFKF1_1 interaction protein-encoding genes were down-regulated in flower buds under white light. Under blue light, only *BjuCOL5*, *BjuSKP1f*, *BjuCOL3*, *BjuAP2*, *BjuAP2-1* and *BjuLKP2* were up-regulated in flower buds. During development, *BjuAP2*, *BjuCOL3*, *BjuCOL5*, *BjuAP2-1* and *BjuAP2* were up-regulated at the reproductive stage under white light, and *BjuCOL, BjuCOL5*, *BjuAP2* and *BjuLKP2* were up-regulated at the reproductive stage under blue light, compared with the wild type. GUS staining assays revealed that *BjuFKF1_1* was expressed in all inflorescences and in both the front and bottom parts of siliques, but no obvious GUS signal was observed in seeds under the detection conditions. This work investigated the role of *BjuFKF1_1* in promoting flowering and offers new insights into accelerating the breeding of *B. juncea.*

## 2. Results

### 2.1. Identification and Physiochemical Characteristics of Photoreceptor Gene Families in Brassica Species

**Figure 1:**
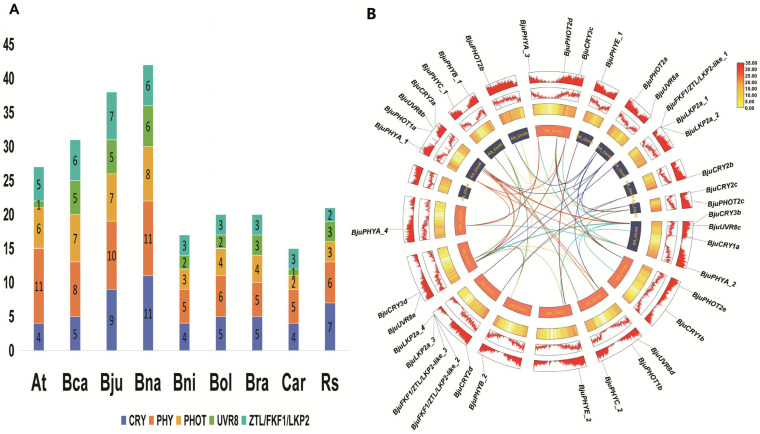
Analysis of evolution of PHRs across nine Brassicaceae species and the distribution of BjuPHRs in *B. juncea.* (**A**) A number of *PHR* gene homologues were identified across nine Brassicaceae species. (**B**) Chromosomal locations of *BjuPHR* genes in *B. juncea* and synteny analysis of inter-chromosomal relationships of *BjuPHR* genes. The multicolored lines indicate segment-duplicated *BjuPHR* gene pairs. Lines are color-coded to represent different duplication events or homologous chromosome relationships.

**Figure 2:**
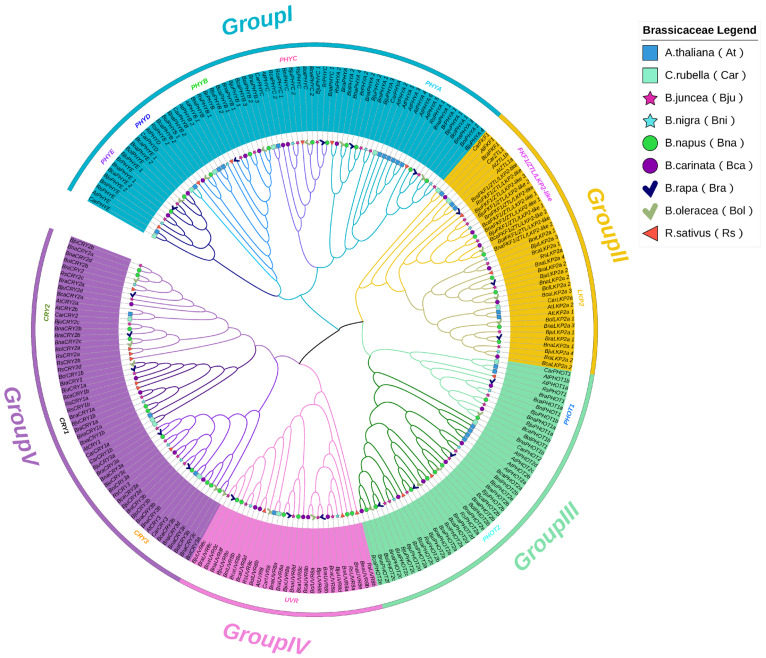
Evolutionary analysis of *BjuPHR* homologues across nine Brassicaceae species. The tree is rooted and categorized into four major clades (Group I–IV), indicating distinct evolutionary lineages. The different colored lines represent the different subgroups in major clades (Group I–IV).

A total of 54 CRYs, 67 PHYs, 44 PHOTs, 29 UVR8s and 38 ZTL/FKF1/LKP2 homologues were identified across nine Brassicaceae species. Among those, *B. napus* (Bna), *B. juncea* (Bju) and *B. carinata* (Bca) presented the greatest numbers of *PHR* genes, followed by *Arabidopsis thaliana* (At), *B. oleracea* (Bol), *B. rapa* (Bra) and *Raphanus sativus* (Rs), whereas *B. nigra* (Bni) and *Capsella rubella* (Cra) presented the lowest numbers, which indicated that *PHR* expansions originated from ancient segmental and tandem duplications in diploid ancestors (Figure 1A). The CRY family, comprising 40 members across nine species, exhibited significant structural diversity. A clear distinction was observed between CRY2 and CRY2-like subfamilies; compared with canonical CRY2 members, CRY2-like proteins were shorter (~575 AA) (~623 AA), resulting in a lower molecular weight and hydrophilic coefficient, while their isoelectric point (pI) ranged from 5.56 to 5.99. Both subgroups were predicted to localize in chloroplasts. In contrast to the dual localization (chloroplast and mitochondria) of AtCRY3, *Brassica* CRY3 proteins were primarily chloroplast-localized, whereas all identified CRY3-like members were predicted to reside in both chloroplasts and mitochondria. The size variation within the *Brassica* CRY family was substantial, with BnCRY3-like_4 (813 AA) being the greatest and BnCRY3-like_1 (516 AA) being the smallest. All *Brassica* PHY members, including newly identified PHYA-like proteins, were consistently predicted to function in the nucleus. They exhibited a highly conserved length of approximately 1131 amino acids and a narrow pI range between 5.55 and 6.29. Similarly, ZTL/FKF1/LKP2 proteins were also localized in the nucleus. Notably, compared with other members of this group, the ZTL/FKF1/LKP2-like subfamily was characterized by a higher theoretical pI. In addition, members of the PHOT family were predominantly predicted to be associated with cell membranes. However, three specific members (BraPHOT1, BjuPHOT1-1 and BjuPHOT2-1) were additionally predicted to localize in the nucleus. The PHOT family showed considerable variation, with an average length of ~911 AA (ranging from 727 to 996 AA) and a broad pI spectrum from 6.19 to 8.7. In contrast to other PHRs, UVR8 members predicted single-to-multiple-site location. The majority were predicted to be nuclear, with a significant number also localizing to the cell wall and cytoplasm. A unique case was BolUVR8-like_3, which was predicted to be present in the Golgi apparatus in addition to the cytoplasm and nucleus (Table S2). In *B. juncea*, the 9 *CRY*, 10 *PHY*, 7 *PHOT*, 5 *UVR8* and 7 *ZTL/FKF1/LKP2* genes were identified and distributed across all chromosomes except A04, with four PHRs localized to AA_chr09, AA_chr06 and BB_chr03 (Figure 1B). Phylogenetic reconstruction with *Arabidopsis* homologues divided PHRs into distinct clades: CRY (3 subfamilies), PHY (5), UVR8 (1), PHOT (2) and ZTL/FKF1/LKP2 (2) (Figure 2, Table S3).

### 2.2. Gene Structure, Motif Discovery and Promoter Analysis

**Figure 3:**
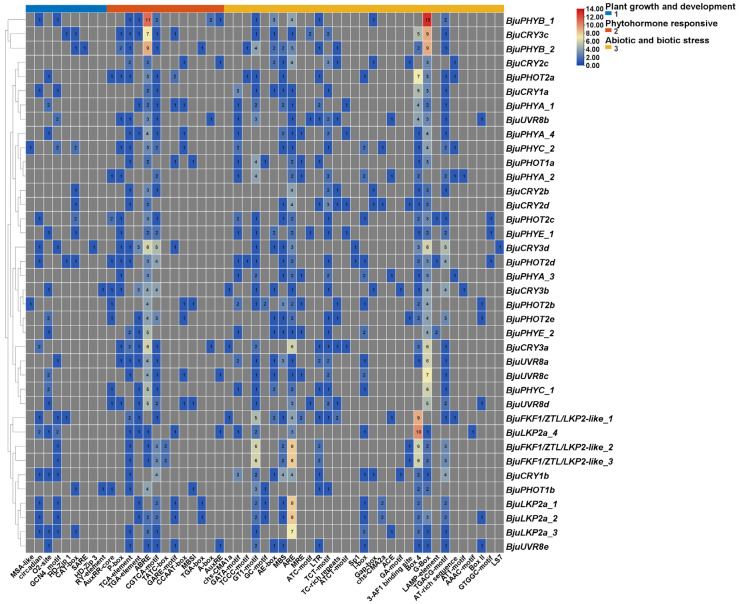
Cis-element analysis of the upstream 2 kb region of *BjuPHR* genes. Different colors represent different types of cis elements. The color intensity and number in the box indicate the numbers of cis elements in these *BjuPHR* genes.

To comprehensively assess evolutionary diversification among *PHR* genes, comparative structural analyses were conducted for two or more genes of each family (*CRY*, *PHY*, *UVR8*, *PHOT* and *ZTL/FKF1/LKP2*). The results revealed that each family (*CRY*, *PHY*, *UVR8*, *PHOT* and *ZTL/FKF1/LKP2*) exhibited conserved exon/intron architectures (number, order and symmetry), relative to those of *Arabidopsis* homologues, confirming the evolutionary conservation of each family (Figure S1). In terms of species, the structural conservation of *B. napus* was the greatest, whereas that of *Capsella rubella* was the lowest. In addition, seven members of *CRY* (*BjuCRY2d*, *BcaCRY2a*, *BRaCRY2a*, *CarCRY1a*, *CarCRY1b*, *BcaCRY3a* and *BnaCRY3e*), four members of *ZTL/FKF1/LKP2* (*BcaFKF1*, *AtLKP2a_1*, *AtFKF1* and *CarFKF1*), *CarPHOT2*, four members of *PHY* (*RsPHYA-2*, *BrPHYA-1*, *CarPHYA* and *AtPHYA-2*) and three numbers of *UVR8* (*RsUVR8b*, *RsUVR8c* and *BnaUVR8d*) were notable exceptions. Moreover, in silico promoter analysis of *B. juncea* PHRs revealed enrichment in light responsiveness (Box-4 and G-Box) and ABA responsiveness (ABRE). Of those, *BjuFKF1/ZTL/LKP2-like_1* (hereafter *BjuFKF1_1*) and *BjuLKP2a_4* were identified as important regulators of light responsiveness (Box-4 motif numbers: 9 and 10, respectively), as were *BjuPHYB_1*, *BjuCRY3c* and *BjuPHYB_2* (G-Box motif numbers: 13, 9 and 9, respectively) and ABA responsiveness (ABRE motif numbers: 11, 7 and 9, respectively), suggesting that those PHRs might play important roles in light responsiveness and ABA responsiveness. The number of development-related motifs was relatively greater in *PHOT* genes than in other *PHR* genes, but the mean number of ABA- and stress-related motifs was markedly greater in the *UVR8* genes (Figure 3, Table S4). These findings indicate that BjuPHRs predominantly regulate plant development through light-responsive pathways.

### 2.3. Profiling of PHR Gene Expression in Different Tissues and Tumorous Stem Developmental Stages

**Figure 4:**
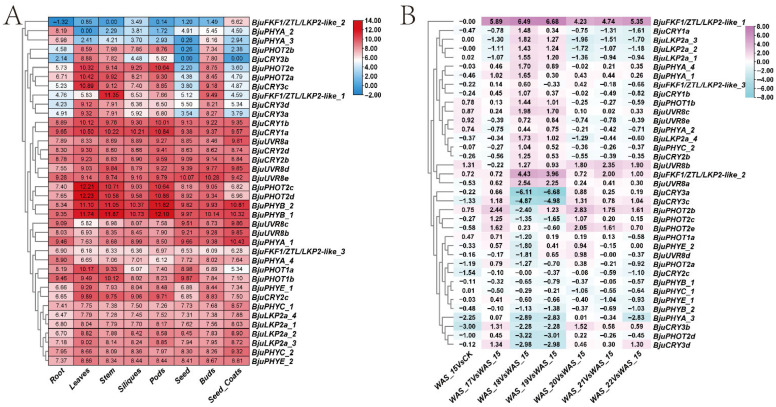
The expression patterns of *BjuPHR* genes during organogenesis and tumorous stem developmental stages in *B. juncea* were determined via RNA-seq and seven candidate PHRs were validated by qRT-PCR in the ‘YA-1’ and ‘ZT-1’ cultivars. (**A**) The expression patterns of *BjuPHR* genes in different organs. (**B**) The expression patterns of *BjuPHR* genes during tumorous stem developmental stages.

**Figure 5:**
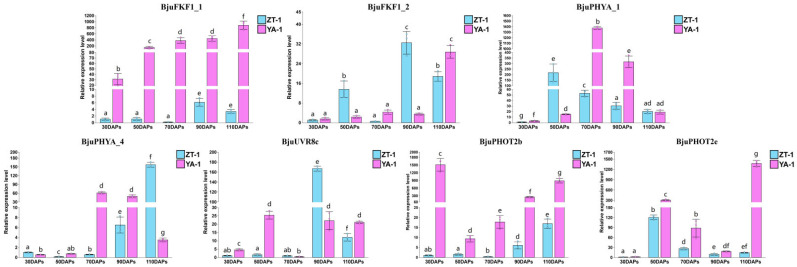
Seven candidate PHRs were validated by qRT-PCR in the ‘YA-1’ and ‘ZT-1’ cultivars. The standard error calculated from three biological replicates and lowercase letters above the bars indicate statistically homogeneous groups as determined by Duncan’s least significant range (LSR) test (*p* < 0.05) following a two-way ANOVA. Within each cultivar and for each gene, bars that share the same letter are not significantly different from one another.

Owing to the presence of several light-associated promoter motifs in *PHR* genes, the RNA-seq-based expression profiles of different organ samples consisting of roots, stems, leaves, buds, siliques, pods, seeds and seed coats, as well as tumorous stem developmental stages (CK, WAS15, WAS17, WAS19, WAS20, WAS21, WAS22 and WAS25) were investigated. Most of these genes (*BjuFKF1_1*, *BjuPHOT1a*, *BjuLKP2a_4*, *BjuLKP2a_1*, *BjuPHYC_1*, *BjuLKP2a_2, BjuPHOT1b*, *BjuLKP2a_3*, *BjuPHYA_4*, *BjuPHYC_2*, *BjuPHYE_1*, *BjuUVR8a*, *BjuCRY2d, BjuPHYE_2*, *BjuUVR8c*, *BjuCRY2c*, *BjuPHOT2c*, *BjuCRY2b*, *BjuCRY1b*, *BjuUVR8b*, *BjuPHOT2d, BjuCRY1a*, *BjuPHYA_1*, *BjuUVR8d, BjuPHYB_2*, *BjuPHYB_1* and *BjuUVR8e)* accumulated in all organ samples. In particular, *BjuFKF1_1*, *BjuCRY1a*, *BjuPHOT2c*, *BjuPHOT2d*, *BjuPHYB_2*, *BjuPHYB_1* and *BjuPHOT1b* were highly accumulated in the stem (Figure 4A, Table S5). In addition, compared with those in WAS15, four transcripts belonging to the *CRY* family (*BjuCRYd3a*, *BjuCRYd3b*, *BjuCRYd3c* and *BjuCRYd3d*) were significantly down-regulated in WAS19 and WAS20. *BjuFKF1_1* was significantly changed from WAS18 to WAS25, followed by *BjuFKF1/ZTL/LKP2-like_2* (*BjuFKF1_2*)*,* whereas *BjuFKF1/ZTL/LKP2-like_3* (*BjuFKF1_3*) was not detected in any tumorous stem developmental stages. *BjuPHOT2b* and *BjuPHOT2e* significantly changed from WAS18 to WAS25, except for WAS19 and WAS20, respectively. In addition, *BjuPHYA1* and *BjuPHYA4* were up-regulated from WAS18 to WAS25. *BjuUVR8b* significantly changed from WAS17 to WAS25, except for WAS18 (Figure 4B, Table S6). To validate the *PHR* genes, whose expression was strongly altered (*BjuFKF1_1*, *BjuFKF1_2*, *BjuPHYA_1*, *BjuPHYA_4*, *BjuUVR8c*, *BjuPHOT2b* and *BjuPHOT2e*), qRT-PCR was conducted on tumorous stem samples collected at 30, 50, 70, 90 and 110 days after planting (DAP), with three biological replicates from bolting-resistant ‘ZT-1’ and bolting-susceptible ‘YA-1’ plants. *BjuFKF1_1* and *BjuPHOT2b* expression levels were significantly more abundant in ‘YA-1’ than in ‘ZT-1’ from 30 DAP to 110 DAP (Figure 5), whereas *BjuFKF1_1* accumulated only from 70 DAP to 110 DAP (Figure 5). *BjuPHOT2e* was significantly more abundant in ‘YA-1’ from 30 DAP to 110 DAP than in ‘ZT-1’, except at 30 DAP (Figure 5). *BjuPHYA1* and *BjuPHYA4* expression levels were more enriched at 70 DAP and 90 DAP in ‘YA-1’ than in ‘ZT-1’, respectively (Figure 5). In addition, compared with ‘ZT-1’, *BjuUVR8b* was more enriched from 30 DAP to 110 DAP, except at 90 DAP (Figure 5). Overall, the qRT-PCR-validated expression patterns results suggesting that *BjuFKF1_1* may play a role in regulating tumorous stem development. Furthermore, *BjuFKF1_1* expression was consistently and significantly higher in ‘YA-1’ than in ‘ZT-1’ from 30 DAP to 110 DAP. Therefore, *BjuFKF1_1* was selected for further functional analysis in *B. juncea*.

### 2.4. Agrobacterium-Mediated BjuFKF1_1 OE Line Screening

**Figure 6:**
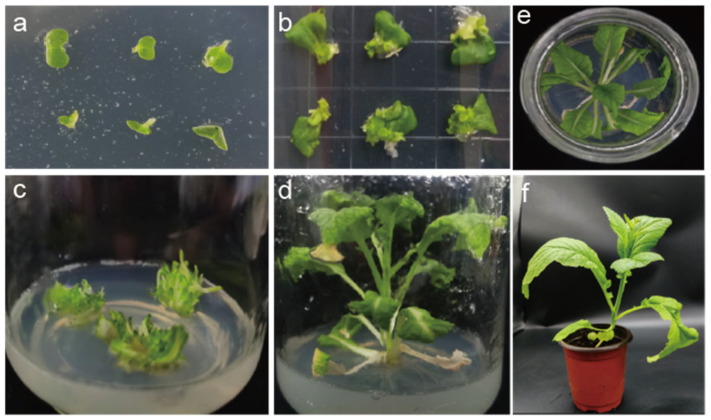
Generation of the *BjuFKF1_1* OE lines. (**a**,**b**) Hygromycin-resistant callus tissue, induced using *Agrobacterium tumefaciens* strain GV3101. (**c**) Hygromycin-resistant adventitious bud regeneration from a callus. (**d**–**f**) Independent hygromycin-resistant transgenic plants established from regenerated buds.

**Figure 7:**
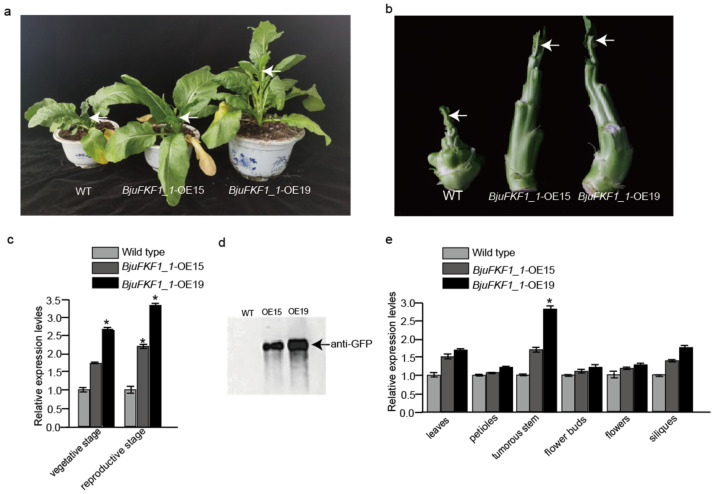
Phenotypic and molecular characterization of *BjuFKF1_1* OE lines. (**a**,**b**) Flowering phenotypes of *BjuFKF1_1* OE line15 and 19, compared with the wild type. (**c**) Relative *BjuFKF1_1* transcript levels in *BjuFKF1_1* OE line15 and 19 at the vegetative and reproductive stages compared with the wild type. (**d**) The BjuFKF1_1-GFPs expression levels in OE15 and OE19. (**e**) *BjuFKF1_1* expression levels in different tissues (leaf, tumorous stem, petiole, flower bud, flower and silique) in *BjuFKF1_1* OE line15 and line19. * *p* < 0.05.

To investigate the function of *BjuFKF1_1* in plant development, *Agrobacterium*-mediated *BjuFKF1_1* OE lines were generated (Figure 6). A total of 20 candidate *BjuFKF1_1* OE lines were obtained and further screened at the genomic DNA (gDNA) level. The gDNAs from three wild types (WT) were used as a control. Amplification of the *green fluorescent protein* (*GFP*) sequence confirmed successful transgene integration in 5 out of 20 independent transgenic lines (Figure S2). The flowering times of the OE15 and OE19 lines were significantly earlier than the wild type in the field, with the flowering time being 54 d after transplanting for line15 and 52 d after transplanting for line19 (Figure 7). However, the flowering day for the wild type is 98 d after transplanting. In addition, qRT-PCR analysis revealed that *BjuFKF1_1* transcript levels were up-regulated >1.5-fold at the vegetative stage and >2-fold at the reproductive stage in OE line15, while they were up-regulated >2.5-fold at the vegetative stage and >3-fold at the reproductive stage in OE line19, compared with WT (Figure 7). Western blot analysis further confirmed the accumulation of the GFP-BjuFKF1_1 protein in OE line15 and 19 (Figure 7d). Moreover, tissue-specific expression analysis revealed that the *BjuFKF1_1* mRNA level was up-regulated 1.5-fold in the leaves and tumorous stems, but remained largely unchanged in the petioles, flower buds, flowers and siliques in OE line15, while it was up-regulated 2.5-fold in the tumorous stems and 1.5-fold in the leaves and siliques but remained largely unchanged in the petioles, flower buds and flowers in OE line 19, compared with WT (Figure 7e).

### 2.5. Expression Profile of BjuFKF1_1

**Figure 8:**
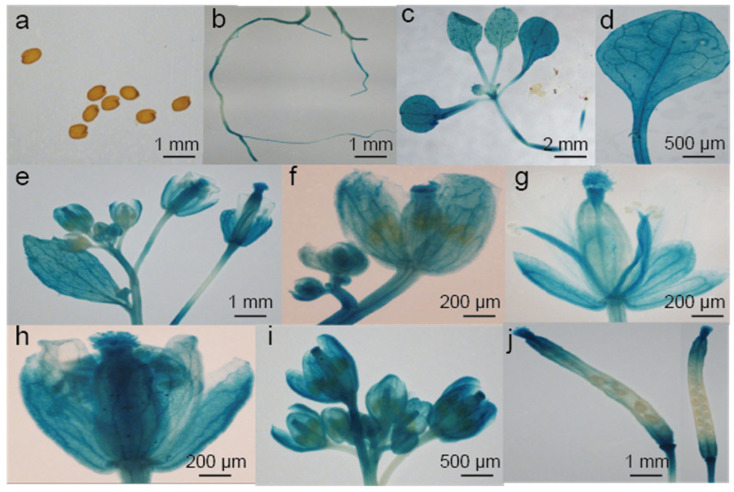
Spatial expression pattern of *BjuFKF1_1* promoter-driven GUS activity in *A*. *thaliana*. (**a**) Seeds. (**b**) Root. (**c**,**d**) Seedlings ((**c**) whole seedling and (**d**) cotyledon and hypocotyl vasculature). (**e**) Inflorescence. (**f**) Sepal. (**g**) Petal. (**h**) Stamen filament and pistil style. (**i**) Silique valves and replum. (**j**) Silique transverse section (absence in seeds).

Consistent GUS staining patterns were observed across more than three independent transgenic lines by examining their GUS activities. Overall, histochemical analysis revealed *BjuFKF1_1* promoter activity in multiple tissues throughout development (Figure 8). The *Arabidopsis* seeds were first checked after 2 d of soaking and no signal was detected (Figure 8a). In the seedling stage, the GUS signal was detected in the roots and the vasculature of cotyledons and leaves (Figure 8b–d). During the reproductive stage, the sepals, petals, filaments, styles and stigma bases were stained (Figure 8e–i). Within the siliques, no obvious GUS signal was observed in seeds under the detection conditions (Figure 8a,j).

### 2.6. Transcriptional Profiling of BjuFKF1_1 Interaction Protein-Encoding Genes

**Figure 9:**
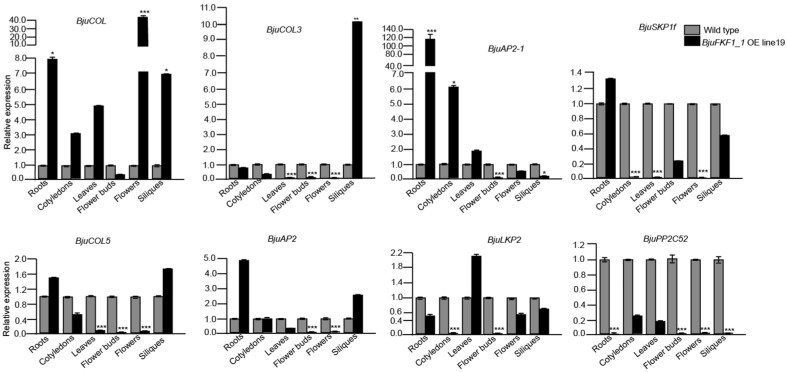
BjuFKF1_1 interaction protein-encoding genes expression levels in different tissues in *BjuFKF1_1* OE line19. * *p* < 0.05, *** *p* < 0.0001.

**Figure 10:**
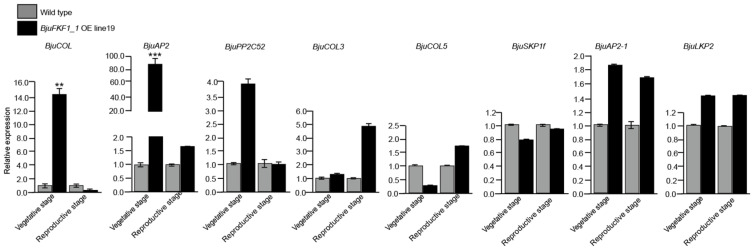
BjuFKF1_1 interacting protein-encoding genes expression levels at different developmental stages in *BjuFKF1_1* OE line19. ** *p* < 0.001, *** *p* < 0.0001.

Changes in the transcription of genes encoding BjuFKF1_1 interaction proteins were analyzed via qRT-PCR in the wild type and *BjuFKF1_1* OE line19 (Figure 9). The results indicated that *BjuCOL* was up-regulated in the roots, cotyledons, leaves, flowers and siliques (significant in flowers and siliques), but was down-regulated in the flower buds; *BjuCOL3* was only up-regulated in the siliques but was down-regulated in the other tissues (significant in leaves, flower buds and flowers); *BjuAP2-1* was up-regulated in the roots, cotyledons and leaves (significant in roots and cotyledons), but was down-regulated in the flower buds, flowers and siliques (significant in flower buds and siliques); *BjuSKP1f* was only up-regulated in the roots, but it was down-regulated in the cotyledons, leaves, flower buds, flowers and siliques (significant in cotyledons, leaves and flowers); *BjuCOL5* was up-regulated in both the roots and siliques, but was down-regulated in the cotyledons, leaves, flower buds and flowers (significant in leaves, flower buds and flowers); *BjuAP2* was up-regulated in both the roots and siliques, but was down-regulated in the leaves, flower buds and flowers (significant in flower buds and flowers), while there was no change in the cytoledons; *BjuLKP2* was only up-regulated in the leaves, while it was down-regulated in the roots, cytoledons, flower buds, flowers and siliques (significant in cotyledons and flower buds); *BjuPP2C52* was down-regulated in all tissues, especially the roots, flower buds, flowers and siliques (Figure 9). During different developmental stages, *BjuCOL*, *BjuAP2*, *BjuPP2C52*, *BjuCOL3*, *BjuAP2-1* and *BjuLKP2* were up-regulated, but *BjuCOL5* and *BjuSKP1f* were down-regulated at the vegetative stage. At the reproductive stage, *BjuCOL*, *BjuPP2C52* and *BjuSKP1f* were down-regulated, while *BjuAP2*, *BjuCOL3*, *BjuCOL5*, *BjuAP2-1* and *BjuLKP2* were up-regulated (Figure 10).

### 2.7. Changes in the Transcription of Genes Encoding BjuFKF1_1 -Interaction Proteins in BjuFKF1_1 OE Line19 Under Blue Light

**Figure 11:**
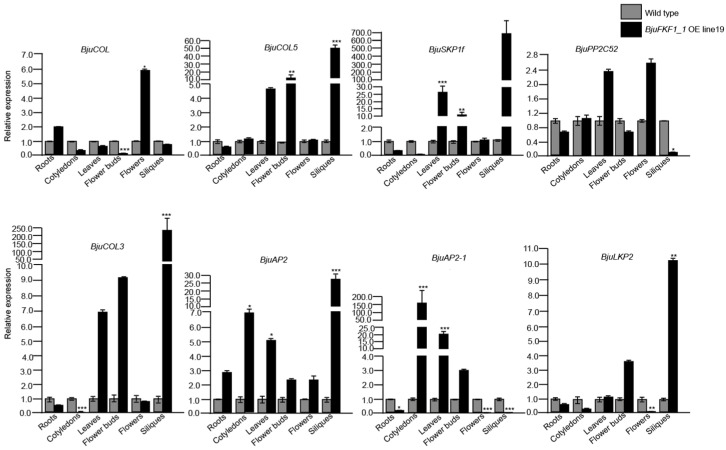
BjuFKF1_1 interaction protein-encoding genes expression levels in different tissues in *BjuFKF1_1* OE line19 under blue light. * *p* < 0.05, ** *p* < 0.001, *** *p* < 0.0001.

**Figure 12:**
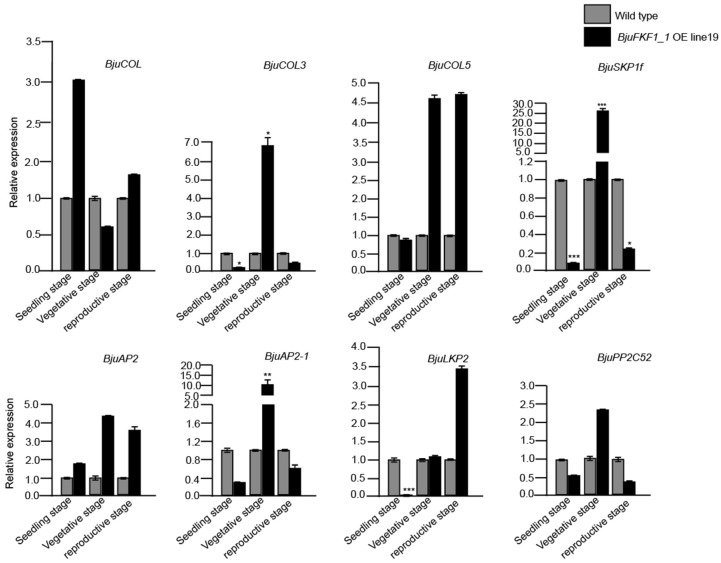
BjuFKF1_1 interaction protein-encoding genes expression levels at different developmental stages in *BjuFKF1_1* OE line19 under blue light. * *p* < 0.05, ** *p* < 0.001, *** *p* < 0.0001.

Given that *FKF1* is a plant-specific blue light receptor, we also detected the transcript levels of BjuFKF1_1 interaction protein-encoding genes in *BjuFKF1_1* OE line19 under blue light (Figure 11). In different tissues, *BjuCOL* increased in the roots and flowers but decreased in cotyledons, leaves, flower buds and siliques. For *BjuCOL5*, it was up-regulated in all aerial tissues, except for the roots, in which it was down-regulated. Similarly, *BjuSKP1f* was up-regulated in the leaves, flower buds, flowers and siliques but was down-regulated in the roots and cotyledons. *BjuPP2C52* expression increased in the cotyledons, leaves and flowers but decreased in the roots, flower buds and siliques. *BjuCOL3* expression was elevated in the leaves, floral buds and siliques but was reduced in the roots, cotyledons and flowers. Notably, *BjuAP2* was universally up-regulated across all tissues and was significantly up-regulated in the cotyledons, leaves and siliques. *BjuAP2-1* expression increased in the cotyledons, leaves and flower buds but decreased in the roots, flowers and siliques. Finally, *BjuLKP2* was up-regulated in the leaves, flower buds and siliques but was down-regulated in the roots, cotyledons and flowers (Figure 11). During the different developmental stages, at the seedling stage, only *BjuCOL* and *BjuAP2* were up-regulated. In contrast, all the other interaction protein-encoding genes (*BjuCOL3*, *BjuCOL5*, *BjuSKP1f*, *BjuAP2-1*, *BjuLKP2* and *BjuPP2C52*) were down-regulated. At the vegetative stage, except *BjuCOL*, all other eight interaction protein-encoding genes were up-regulated. However, at the reproductive stage, the expression of the genes *BjuCOL*, *BjuCOL5*, *BjuAP2* and *BjuLKP2* were increased, whereas *BjuCOL3*, *BjuSKP1f*, *BjuAP2-1* and *BjuPP2C52* were decreased (Figure 12).

## 3. Discussion

On the basis of our previous study, a comparative analysis between *B. juncea* (*Bju*) and *A. thaliana* (*At*), focusing on *FKF1*-mediated blue light signaling pathways was performed. *FKF1*, a plant-specific LOV-domain blue light receptor, has conserved flowering promoting functions across plant species [36]. However, our comparative analysis revealed significant mechanistic differences between *A. thaliana* and *B. juncea* (Figure 1 and Figure 2). In *Arabidopsis*, the FKF1-GI-CDF1 and FKF1-COP1 modules integrate photoperiodic signals to regulate *CO* expression, thereby modulating the flowering initiation gene *FT* [42,43,50,51,52,53]. While *AtFKF1* expression was previously detected in sepals and anther filaments, histochemical GUS staining in this study revealed that *BjuFKF1_1* was also expressed in inflorescences, including sepals, petals, filaments, styles and the base of the stigma (Figure 8) [36]. Consistent with its proposed flowering-promoting function, the *BjuFKF1_1* OE lines exhibited significantly early flowering in the field, which can be further used as a negative molecular marker or a target gene for bolting-resistant variety selection (Figure 7). Notably, flowering time regulators such as TOE interact with the CO and COL transcription factors to suppress *CO* activity and delay flowering [54]. Furthermore, while *CO* and *COL3* promote flowering under long-day conditions, *COL5* functions preferentially under short-day conditions [55,56]. Given that BjuFKF1_1 physically interacts with BjuCOL, BjuCOL3 and BjuCOL5, we detected the transcriptional levels of these genes in the *BjuFKF1_1* OE background [49]. Under white light, only *BjuCOL* was up-regulated in flowers and both *BjuCOL3* and *BjuCOL5* were up-regulated at the reproductive stage, compared to the wild type (Figure 9 and Figure 10). When under blue light, *BjuCOL* was up-regulated in flowers but both *BjuCOL* and *BjuCOL5* were up-regulated at the reproductive stage compared to the wild type (Figure 11 and Figure 12). Collectively, these results demonstrate that blue light selectively modulates the transcript accumulation of *BjuCOL* family genes in a *BjuFKF1_1*-dependent manner. *BjuFKF1_1* overexpression significantly altered the expression of *BjuCOL* family genes in a blue-light-dependent manner.

*AP2* family genes play crucial roles in regulating plant developmental processes. *AtAP2*, the first identified *AP2-like* gene, functions in floral meristem establishment, floral organ identity and ovule and seed development [57,58,59]. Given that BjuFKF1_1 physically interacts with both BjuAP2 and BjuAP2-1, we examined their expression patterns in the *BjuFKF1_1* OE background [49]. Under white light, both *BjuAP2* and *BjuAP2-1* were down-regulated in flowers but both of them were up-regulated at the reproductive stage, compared with the wild type (Figure 9 and Figure 10). In contrast, under blue light, *BjuAP2* expression was up-regulated in flowers and at the reproductive stage; however, *BjuAP2-1* was down-regulated in both flowers and at the reproductive stage, compared with the wild type (Figure 11 and Figure 12). These results indicate that blue light specifically modulates *BjuAP2-1* transcript accumulation in a *BjuFKF1_1*-dependent manner. In *Arabidopsis*, *SKP1* functions within the SCF ubiquitin ligase complex by mediating substrate recognition and proteasomal degradation [60]. Here, we observed that *BjuSKP1f* was down-regulated in flowers and at the reproductive stage under white light in the *BjuFKF1_1* OE line (Figure 9 and Figure 10). Under blue light, *BjuSKP1f* was significantly down-regulated at the reproductive stage, but there was almost no change in the flowers, compared to the wild type (Figure 11 and Figure 12). These findings demonstrate that blue light strongly influences *BjuSKP1f* expression in a *BjuFKF1_1* OE background, suggesting a potential role for light quality in regulating the SKP1-mediated ubiquitination pathway.

*LKP2*, another plant blue light receptor, has been reported to delay flowering under short-day conditions [37]. Our previous work demonstrated that BjuFKF1_1 interacts with BjuLKP2, specifically via its respective F-box domain [49]. In *BjuFKF1_1* OE line19, *BjuLKP2* was down-regulated in the flowers, but it was up-regulated at the reproductive stage under white light (Figure 9 and Figure 10); under blue light, *BjuLKP2* was significantly down-regulated in flowers, but it was up-regulated at the reproductive stage in *BjuFKF1_1* OE line19 (Figure 11 and Figure 12). This indicates that *BjuLKP2* transcript accumulation is not primarily modulated by blue light in this genetic background. Previous studies have shown that both BjuFKF1_1 and BjuLKP2 interact with BjuPP2C52 [48,49]. In the present study, *BjuPP2C52* expression was significantly down-regulated in flowers and there was almost no change at the reproductive stage under white light, compared with the wild type (Figure 9 and Figure 10). In contrast, under blue light, it was up-regulated in the flowers (Figure 11). During development, *BjuPP2C52* was down-regulated at the reproductive stage under blue light conditions (Figure 11 and Figure 12). These results demonstrate that, unlike that of *BjuLKP2*, the mRNA expression of *BjuPP2C52* is clearly regulated by blue light in *BjuFKF1_1* OE line19.

The findings demonstrate that *BjuFKF1_1* promotes flowering in *B. juncea*. qRT-PCR analysis revealed that while all BjuFKF1_1 interacting protein-encoding genes were down-regulated in flowers under white light conditions, *BjuCOL*, *BjuCOL5*, *BjuSKP1f*, *BjuPP2C52* and *BjuAP2* were significantly up-regulated in flowers under blue light. Furthermore, developmental stage analysis revealed that *BjuAP2*, *BjuCOL3*, *BjuCOL5, BjuAP2-1* and *BjuLKP2* were up-regulated at the reproductive stage in *BjuFKF1_1* OE line19 compared with the wild type. Notably, under blue light, *BjuCOL*, *BjuCOL5*, *BjuAP2* and *BjuLKP2* were up-regulated at the reproductive stage. In support of these findings, GUS staining assays revealed *BjuFKF1_1* expression in the seedlings, leaves, flower buds and siliques. Taken together, the results of this study reveal that *BjuFKF1_1* promotes flowering in *B. juncea*. It accelerates flowering through photoperiodic regulation of flowering-time genes in *B. juncea*.

## 4. Materials and Methods

### 4.1. Genome-Wide Analysis of Photoreceptor Gene Families in Brassica Species

The genomic resources of *Brassica juncea* (Bju 2.0), *Brassica napus* (Bna), *Brassica nigra* (Bni), *Brassica rapa* (Bra), *Brassica oleracea* (Bol), *Brassica carinata* (Bca) and *Capsella rubella* (Car) are available at BRAD (http://brassicadb.cn/) accessed on 15 October 2024 and those of *Raphanus sativus* (Rs) are available in the radish genome database (https://radish.kazusa.or.jp/) accessed on 15 October 2024. The reference protein sequences of eight *Brassica* species were concatenated (db.fasta) to a complete protein database (makeblastdb -in db.fasta -dbtype prot -out db) to facilitate local BLASTP with *Arabidopsis* query sequences. The protein sequences of five major *PHR* (*photoreceptor*) gene families, namely phytochrome (AtPHYA_NP_172428.1; AtPHYB_ NP_001325249.1; AtPHYC_NP_198433.1; AtPHYD_NP_193360.1; AtPHYE_NP_193547.4), cryptochrome (AtCRY1_NP_567341.1; AtCRY2_NP_849588.1; AtCRY3_NP_568461.3), phototropin (AtPHOT1_NP_190164.1; AtPHOT2_NP_001318824.1), F-box containing flavin binding proteins (AtZTL_NP_568855.1; AtFKF1_NP_564919.1; AtLKP2_NP_849983.1) and UV RESISTANCE LOCUS 8 (AtUVR8_NP_201191.1) were used as queries for the identification of *Brassica PHY*, *CRY*, *PHOT*, *ZTL/FKF1/LKP2* and *UVR8* gene family members, respectively (blastp -query.fa -db -outfmt 6 -evalue 1 × 10^−10^ -out target.fa -max_target_seqs 20-num_threads 4). After that, the best BLAST hits were further validated for their characteristic conserved domains of the respective gene family via CD-search tool [61]. Several bioinformatics tools, including ProtParam (http://web.expasy.org/protparam) accessed on 18 October 2024, WoLF PSORT (https://wolfpsort.hgc.jp/) accessed on 18 October 2024 and TMHMM Server v2.0 (http://www.cbs.dtu.dk/services/TMHMM/) accessed on 18 October 2024 were used to analyze the physical and chemical properties of the confirmed proteins [62,63,64]. For cis-regulatory element analysis, a 2 kb promoter region upstream of each BjuPHRs gene was extracted by using the “GXF Sequence Extract” tool implemented in TBtool and analyzed using the PlantCARE database, and the results were visualized as a heatmap with TBtools-II [65,66,67].

### 4.2. Phylogenetic, Motif and Structural Prediction of BjuPHRs in B. juncea

To classify the evolutionary relationships of BjuPHRs proteins, sequences from nine Brassicaceae species were retrieved from the Brad 3.0 database (http://www.brassicadb.cn/#/) accessed on 15 December 2024. An un-rooted phylogenetic tree was constructed on the basis of aligned protein sequences using the maximum likelihood method implemented in MEGA11 with 1000 bootstrap replicates [68]. The resulting tree was subsequently visualized using an interactive iTOL V7 web platform (https://itol.embl.de/) accessed on 25 December 2024 [69]. To gain insight into the structural diversity of BjuPHRs genes, their exon-intron organizations were analyzed with TB tools. In parallel, conserved protein motifs were identified via the MEME Suite 5.5.9 online tool (https://meme-suite.org/meme/tools/meme) accessed on 28 December 2024, with the maximum number of motifs set to 10. In addition, for cis-regulatory element analysis, a 2 kb promoter region upstream of each BjuPHRs gene was extracted using the “GXF Sequence Extract” tool implemented in TBtool and analyzed using the PlantCARE database (http://bioinformatics.psb.ugent.be/webtools/plantcare/html/) accessed on 18 October 2024, and the results were visualized as a heatmap with TBtools-II [65,66].

### 4.3. Analysis of RNA-Seq Data for Organ and Tumorous Stem Development and Identification of the Expression of Photoreceptor Gene Families

To comprehensively investigate the photoreceptor gene families involved in organ development in *B. juncea*, different organ samples consisting of roots (SRR11787778), stems (SRR11787777), leaves (SRR11787776), buds (SRR11787782), siliques (SRR11787783), pods (SRR11787780), seeds (SRR11787781) and seed coats (SRR807368) were downloaded using iseq software on a Linux system [70]. In addition, the tumorous stem development stages (SRR7427606, SRR7427601 and SRR7427605 for 15 weeks after seeding (WAS15), SRR7427600, SRR7427602 and SRR7427599 for 17 weeks after seeding (WAS17), SRR380275 for 18 weeks after seeding (WAS18), SRR7427603, SRR7427598 and SRR7427604 for 19 weeks after seeding (WAS19), SRR7427596, SRR7427597 and SRR7427595 for 20 weeks after seeding (WAS20), SRR380276 for 21 weeks after seeding (WAS21), SRR380277 for 22 weeks after seeding (WAS22) and SRR380278 for 25 weeks after seeding (WAS25)) were also collected [71]. For consistency, all RNA-seq data were processed according to the methodology described by Kang et al. [66]. Simply, flexbar 3.0 software was employed to extract clean reads from the fastq file by filtering out low-quality reads (>80% of Q30) and removing low-quality sequencing primers and adaptors with the default parameters [72]. Then, HISAT 2.2.1 software was used to align the clear reads to *B. juncea* V2.0 as ref. [73]. The fragments per kilobase of transcript per million mapped reads (FPKM) method was employed to determine the expression level of all global genes and Python 3.7 package HTSeq-Count (HTSeq 2.0.9) was chosen to acquire the number of reads for each gene [74]. Furthermore, R package DESeq2_1.50.1 was employed to identify DEGs [75]. A false discovery rate (FDR) of ≦0.01 and an absolute value of log_2_ Ratio ≦ 1 were selected to assess the expression level of genes [68]. Subsequently, log_2_ (FPKM + 1) values were calculated and averaged with replicates. Finally, a heatmap was generated using TBtools to visualize the expression patterns of BjuPHRs genes [65].

### 4.4. RNA Isolation and qRT-PCR Expression Analysis

In 2024, experiments were conducted in glasshouses at Lishui University (Lishui, China) under natural conditions (18–23 °C). Two *B. juncea* cultivars, namely bolting-resistant ‘ZT-1’ and bolting-susceptible ‘YA-1’ were selected. Tumorous stem samples were collected at 30, 50, 70, 90 and 110 days after planting (DAP), with three biological replicates per cultivar, and stored at −80 °C for RNA extraction. On the basis of results from RNA-seq datasets, *PHR* genes whose expression were strongly altered (*BjuFKF1_1*, *BjuFKF1_2*, *BjuPHYA_1*, *BjuPHYA_4*, *BjuUVR8c*, *BjuPHOT2b* and *BjuPHOT2e*) were selected for qRT-PCR validation and tumorous stem samples, with three biological replicates from ‘ZT-1’ and ‘YA-1’ varieties. For comparisons across multiple growth stages, two-way analysis of variance (ANOVA) was employed, followed by multiple comparisons using Duncan’s least significant range (LSR) tests in SPSS 10.0 statistical software. We performed a variance analysis to evaluate the differences in relative expression levels of genes across various samples, with different letters denoting significant differences at the *p* = 0.05 level. Specifically, the presence of a letter (a, b, c, d, e, f, or g) indicated a significant difference from a column lacking a common superscript letter (*p* < 0.05). Conversely, columns labeled with the same letter were not significantly different at the 5% level, based on Duncan’s multiple range test. To further characterize *BjuFKF1_1*’s function, overexpression lines were generated. The plants were grown under natural field conditions (18–23 °C; 8 h photoperiod/16 h dark). For blue light treatments, plants were maintained at 23 °C under blue LED illumination (16 h light/8 h dark, 5000 LUX). Leaves, petioles, tumorous stems, flower buds, flowers and siliques from wild type and transgenic lines were frozen in liquid nitrogen and stored at −80 °C for RNA extraction. The primers used were designed using Primer premer 5.0, with *BjuActin2* used as an internal reference (Table S1). qRT-PCR was performed on an ABI7500 system (Applied Biosystems, Waltham, MA, USA), according to Zheng et al. (2023), using triplicate technical replicates [76]. The expression levels were quantified as fold changes via the relative quantitative approach (2^−ΔΔCT^) [77].

### 4.5. Generation of BjuFKF1_1 OE Line, Phenotypic Observation and Transcriptional Profiling of BjuFKF1_1 Interaction Protein-Encoding Genes

*BjuFKF1_1* OE lines were generated using the *Agrobacterium tumefaciens*-mediated transformation modified from Jing et al. (2024) [78]. Cotyledon explants of *B. juncea* “yonganxiaoye” were subsequently inoculated with the *A. tumefaciens* strain GV3101 (OD600 = 0.01) harboring p1300-BjuFKF1_1-GFP plasmid for 8 min. After inoculation, explants were co-cultured on an MS medium supplemented with 3 mg/L 6-BA, 0.5 mg/L NAA and 0.8% agar (*w*/*v*) in the dark for 36 h. The tissue was subsequently transferred to different media as follows: (1) Callus induction medium: MS + 3 mg/L 6-BA + 0.5 mg/L NAA + 20 mg/L hygromycin + 500 mg/L Timentin + 0.8% agar; (2) shoot induction medium: MS + 3 mg/L 6-BA + 0.5 mg/L NAA + 20 mg/L hygromycin + 500 mg/L Timentin + 0.8% agar; (3) rooting medium: MS + 0.2 mg/L NAA + 20 mg/L hygromycin + 500 mg/L Timentin + 0.8% agar. Regenerated plantlets were acclimatized in nutrient soil and self-pollinated for three generations. Genomic DNA was isolated from the wild type and *BjuFKF1_1* OE lines, using the CTAB method. The total RNA was extracted from the leaves, petioles, tumorous stems, flower buds, flowers and siliques of the wild type and *BjuFKF1_1* OE line15 and line19, using an RNAprep Pure Plant Kit (Tiangen, Beijing, China). First-strand cDNA was synthesized from 1 µg of RNA with a PrimeScript RT Reagent Kit (Takara Bio, Shiga, Japan). qRT-PCR amplification was performed in triplicate, using SYBR Premix Ex Taq (Takara Bio) on an ABI 7500 system (Applied Biosystems, Waltham, MA, USA) with the following program: 40 cycles of 95 °C for 30 s; 95 °C for 10 s; 58 °C for 10 s; and 72 °C for 10 s. In addition, using a designated extraction buffer (Tris-HCl, pH 7.5, 50 mM; NaCl, 100 mM; EDTA, pH 8.0, 1 mM; and glycerol, 10%; SDS, 0.5%), total protein extraction from p1300- BjuFKF1_1-GFP T_3_ transgenic plants was carried out and Western blot analyses were conducted by utilizing a GFP antibody (ABMART, Berkeley Heights, NJ, USA), with the total protein from the *B. juncea* ‘yonganxiaoye’ wild type serving as the control. Target transcripts (*BjuLKP2*, *BjuPP2C52*, *BjuCOL*, *BjuCOL3*, *BjuCOL5*, *BjuAP2*, *BjuAP2-1* and *BjuSKP1f*) were amplified using gene-specific primers (Table S1). The expression values were normalized to those of *BjuActin2* and calculated using the 2^−ΔΔCT^ method [77]. To assess the flowering time, T_3_ *BjuFKF1_1* OE lines were transplanted to field conditions for natural environmental evaluation and were subsequently grown in controlled environment chambers under white light (400–700 nm) or blue light (450 nm) for photoperiod response analysis.

### 4.6. GUS Staining Assays

The expression pattern of *BjuFKF1_1* was analyzed using transgenic lines expressing the β-glucuronidase (GUS) reporter gene driven by a 2 kb *BjuFKF1_1* promoter fragment. T_1_ transgenic *Arabidopsis* lines were verified by PCR. Positive plants were advanced to the T_2_ generation for segregation analysis. For further analysis, homozygous T_3_ plants were selected on MS media supplemented with 20 mg/L hygromycin for promoter expression studies. Histochemical GUS staining was performed by incubating tissues in staining solution [1 mM/L 5-bromo-4-chloro-3-indolyl β-d-glucuronide, 50 mM/L NaPO_4_ (pH 7.0), 0.4 mM/L K_3_Fe (CN)_6_ and 0.1% (*v*/*v*) Triton X-100] at 37 °C for 6 h. After GUS staining, the chlorophyll was cleared in 70% ethanol for 4 h [79]. Finally, images were acquired using an Olympus MVX10 stereomicroscope (Olympus, Tokyo, Japan).

## Data Availability

The original contributions presented in this study are included in the article/supplementary material. Further inquiries can be directed to the corresponding authors.

## Supplementary Materials

The following supporting information can be downloaded at: https://www.mdpi.com/article/10.3390/plants15020270/s1. Figure S1. Structural analysis of *PHR* genes in *Brassica* species. (A) The phylogenetic tree was constructed on the basis of the full-length sequences of *PHR* genes in *Brassica* species proteins using MEGA 11 software. (B) Each *PHR* gene domain is highlighted by pink, green and yellow boxes. (C) Exon—intron structure of *PHR* genes in *Brassica* species. The green boxes indicate untranslated 5′- and 3′-regions, the yellow boxes indicate exons, and the black lines indicate introns. Figure S2. Amplification of the *green fluorescent protein* (*GFP*) sequence confirmed successful transgene integration in 5 out of 20 independent transgenic lines. M: marker, W1: wild type 1, W2: wild type 2, W3: wild type 3, 1–20: 1–20 *BjuFKF1_1* overexpression lines. M: marker; W1–W3: wild type lines 1–3; 1–20: *BjuFKF1_1* overexpressing lines 1–20. Table S1. List of primers for qRT-PCR analysis, overexpression line construction and GUS staining Table S2. Characterization of *PHR* family genes identified in *Brassica* species. Table S3. Protein sequences of nine *Brassica* species for phylogenetic tree and tree file generation. Table S4. Prediction of cis-regulatory elements in the promoter regions of the *BjuPHR* gene family Table S5. Expression profiles of *BjuPHR* genes in different organs from the available RNA expression profile data. Table S6. Expression profiles of *BjuPHR* genes involved in dynamic stem development from the available RNA expression profile data.
