# Supplementary material for: BjuFKF1_1, a Plant-Specific LOV Blue Light Receptor Gene, Positively Regulates Flowering in Brassica juncea
Source: Plants (Basel). 2026 Jan 15;15(2):270. doi: 10.3390/plants15020270 (PMC12844926; doi:10.3390/plants15020270)
Supplement: Supplementary file 1 [file plants-15-00270-s001.zip › Supplementary Figures and Tables legends.pdf]

Supplementary Figures and Tables legends

Figure S1. Structural analysis of *PHR* genes in *Brassica* species. (A) The phylogenetic tree was constructed on the basis of the full-length sequences of *PHR* genes in *Brassica* species proteins using MEGA 11 software. (B) Each *PHR* gene domain is highlighted by pink, green and yellow boxes. (C) Exon–intron structure of *PHR* genes in *Brassica* species. The green boxes indicate untranslated 5′- and 3′-regions, the yellow boxes indicate exons, and the black lines indicate introns.

Figure S2. Amplification of the *green fluorescent protein* (*GFP*) sequence confirmed successful transgene integration in 5 out of 20 independent transgenic lines. M: marker, W1: wild type 1, W2: wild type 2, W3: wild type 3, 1-20: 1-20 *BjuFKF1_1* overexpression lines. M: marker; W1-W3: wild type lines 1-3; 1-20: *BjuFKF1_1* overexpressing lines 1-20.

Table S1. List of primers for qRT-PCR analysis, overexpression line construction and GUS staining

Table S2. Characterization of *PHR* family genes identified in *Brassica* species

Table S3. Protein sequences of nine *Brassica* species for phylogenetic tree and tree file generation

Table S4. Prediction of cis-regulatory elements in the promoter regions of the *BjuPHR* gene family

Table S5. Expression profiles of *BjuPHR* genes in different organs from the available RNA expression profile data

Table S6. Expression profiles of *BjuPHR* genes involved in dynamic stem development from the available RNA expression profile data
