# Supplementary figures and images for: BjuFKF1_1, a Plant-Specific LOV Blue Light Receptor Gene, Positively Regulates Flowering in Brassica juncea
Source: Plants (Basel). 2026 Jan 15;15(2):270. doi: 10.3390/plants15020270 (PMC12844926; doi:10.3390/plants15020270)

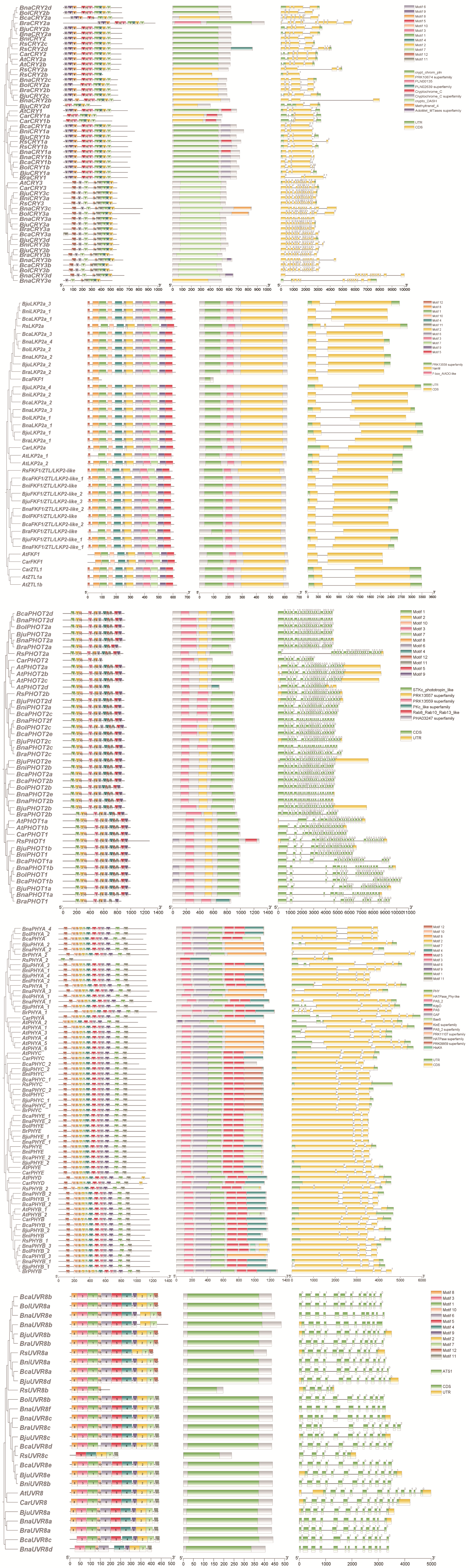

Supplement: Supplementary file 1 [file plants-15-00270-s001.zip › Figure S1.tif]

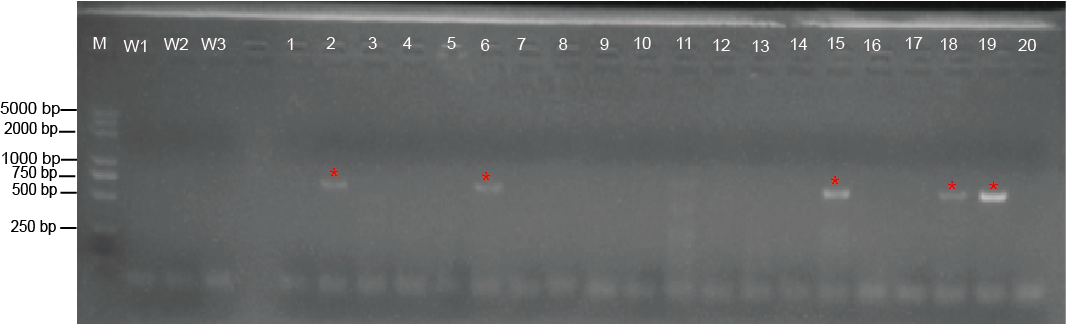

Supplement: Supplementary file 1 [file plants-15-00270-s001.zip › Figure S2.png]
